# Unbiased transcriptome mapping and modeling identify candidate genes and compounds of osteoarthritis

**DOI:** 10.3389/fphar.2022.888533

**Published:** 2022-08-10

**Authors:** Hui Cao, Yifan Fu, Zhenzhen Zhang, Weichun Guo

**Affiliations:** ^1^ Department of Orthopedics, Renmin Hospital of Wuhan University, Wuhan, China; ^2^ The First Clinical School, Tongji Medical College, Huazhong University of Science and Technology, Wuhan, China; ^3^ Department of Rehabilitation, Hankou Hospital, Wuhan, China

**Keywords:** osteoarthritis, CMap, trascriptome, taget genes, compound

## Abstract

Osteoarthritis (OA) is a chronic degenerative joint disease characterized by progressive cartilage loss, subchondral bone remodeling, and synovial inflammation. Given that the current therapies for advanced OA patients are limited, the understanding of mechanisms and novel therapies are urgently needed. In this study, we employed the weighted gene co-expression network (WGCNA) method and the connectivity map (CMap) database to identify the candidate target genes and potential compounds. Four groups of co-expressing genes were identified as the OA-related modules. The biological annotations of these modules indicated some critical hallmarks of OA and aging, such as mitochondrial dysfunctions and abnormal energy metabolism, and the signaling pathways, such as MAPK, TNF, and PI3K/Akt signaling pathways. Some genes, such as *RELA* and *GADD45B*, were predicted to extensively involve these critical pathways, indicating their potential functions in OA mechanisms. Moreover, we constructed the co-expressing networks of modules and identified the hub genes based on network topology. GADD45B, MAFF, and MYC were identified and validated as the hub genes. Finally, anisomycin and MG-262 were predicted to target these OA-related modules, which may be the potential drugs for OA therapy. In conclusion, this study identified the significant modules, signaling pathways, and hub genes relevant to OA and highlighted the potential clinical value of anisomycin and MG-262 as novel therapies in OA management.

## Introduction

As one of the leading causes of disability in the adult population (Altman et al., 2015), osteoarthritis (OA) is a chronic degenerative joint disease, which features a progressive deterioration of cartilage degradation, subchondral bone sclerosis, and synovial inflammation (Kraus et al., 2015). It was estimated in 2019 that ∼ 7% of the global population, more than 500 million people worldwide, are subjected to OA (Mahmoudian et al., 2021). In the southwest region of China, OA affected 13.7% of people in 2012 (Tang et al., 2016). The number of people suffering from OA increases possibly owing to population aging and obesity (Zhang and Jordan, 2010). It was estimated in 1996 that 40% of people aged 70 or older suffer from OA (Valdes and Spector, 2011).

A batch of therapies has been applied in the clinical treatments for OA, which can be classified into three main categories: non-pharmacological methods, pharmacological methods, and surgery (Liu et al., 2018). In the non-pharmacological methods, changing lifestyle and reducing loading on the damaged joint are recommended (Zhang et al., 2008). There are two main options for medication therapies, which are the drugs for treating symptomatic pain and intra-articular (IA) injection. Analgesics (i.e., paracetamol), nonsteroidal anti-inflammatory drugs (NSAIDs) (i.e., meloxicam, diclofenac, and naproxen), specific cyclooxygenase (COX)-2 inhibitors (i.e., celecoxib), and opioids are utilized for systemic drug therapy (Maudens et al., 2018). Patients with hip or knee OA can be treated by IA injections of corticosteroids (i.e., dexamethasone) (Douglas, 2012). However, the efficacy of non-pharmacological therapies is moderated (Siebelt et al., 2014), and the pharmacological treatments are accompanied by several side effects and frequent administration (Maudens et al., 2018). If these treatments fail to relieve pain and improve joint function, joint replacement surgery should be considered (Zhang et al., 2008), while the expenses of surgeries bring a large economic burden for patients. In this context, the understanding of OA initiation and progress as well as the novel targeted therapy are urgently needed. Although emerging studies are focused on the cell and tissue of OA joints (Siebelt et al., 2014), the mechanisms lack cognition. In particular, the lucid molecular mechanisms are still under elucidation (Yang et al., 2018).

Weighted gene co-expression network analysis (WGCNA) can assess the connection of different genes and the potential interactions between them (Langfelder and Horvath, 2008). The highly associated genes are classified as a module. The co-expressing genes assigned to a module tend to have high connectivity. WGCNA can be applied to calculate the correlation of genes across microarray samples, to summarize modules of highly associated genes and key hub genes in the modules, and to relate gene modules to sample traits or other modules. Compared with the other bioinformatics analysis methods, WGCNA preponderates in data processing and gene clustering by a weighted correlation network (Li et al., 2020; Yin et al., 2021).

The connectivity map (CMap) utilizes gene expression profiles to build the association among genes, diseases, and drugs by comparing the gene expression profiles of the human cell lines intervened by more than 1,300 smaller molecules and drugs to the gene signature of the phenotype using a simple yet efficient pattern-matching algorithm (Lamb et al., 2006; Qu and Rajpal, 2012). Potential drugs for diseases were screened by the “connectivity scores” that reflect the connection between the expression profiles or between the drugs and disease (Qu and Rajpal, 2012).

Based on a gene microarray dataset, this study analyzed 34,756 genes from 38 samples by WGNCA, aiming to forecast the significant modules and potential hub genes involved in OA pathogenesis. Since these genes and modules are likely to be biomarkers of OA, these findings might provide some clues to illuminate the molecular mechanisms of OA and develop an improved treatment for OA patients.

## Materials and methods

### Microarray data and preprocessing

**FIGURE 1:**
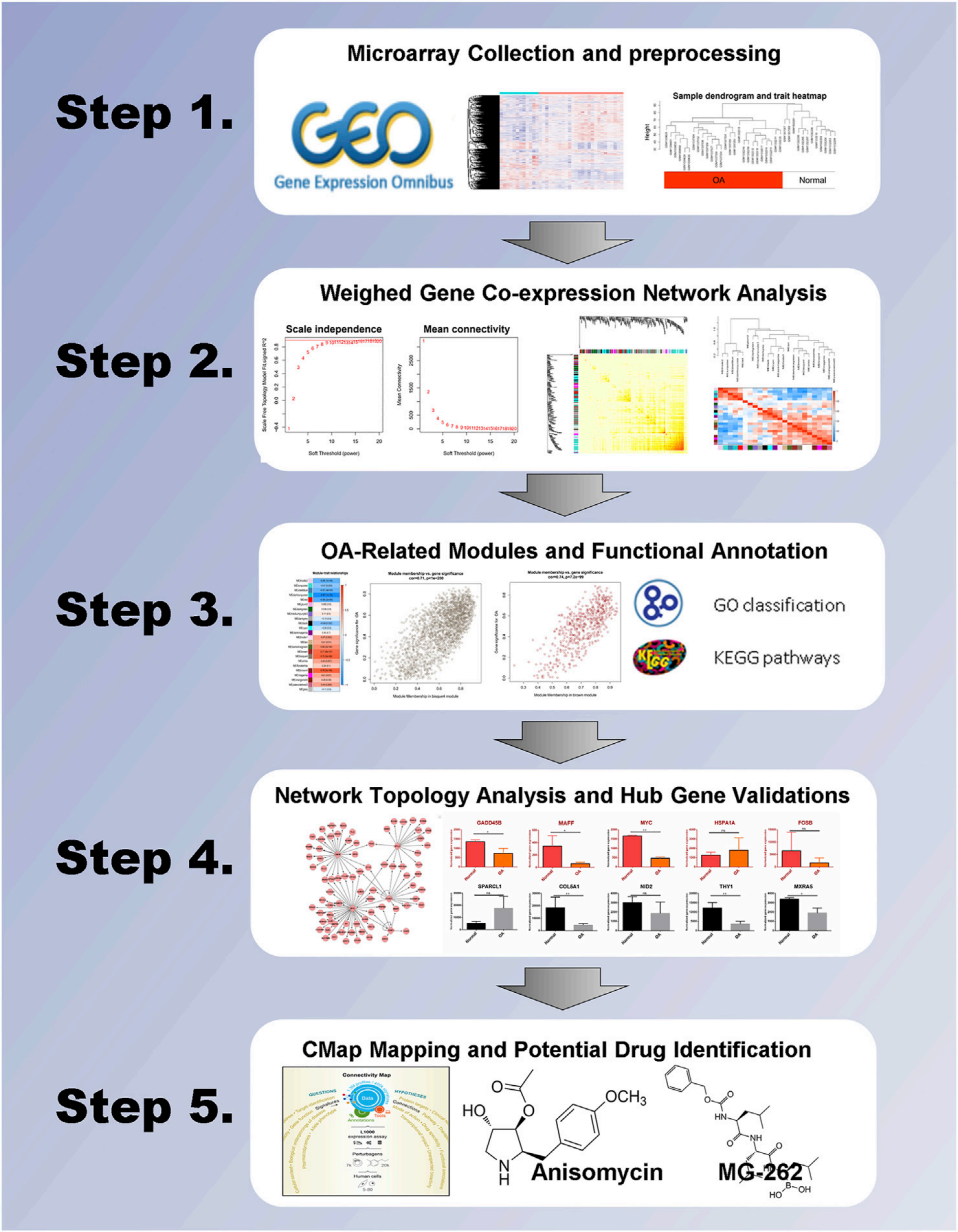
Overview of this study.

The analysis flow chart is summarized in Figure 1. The raw data of microarray-based gene expression with the accession number of GEO: GSE55235, GEO: GSE55457, and GEO: GSE55584 were downloaded from NCBI Gene Expression Omnibus (GEO) on the platform of GPL96 ([HG-U133A] Affymetrix Human Genome U133A Array) (Woetzel et al., 2014). These genome-wide transcriptomic data sets contain the synovial tissues from 20 healthy individuals and 26 patients with osteoarthritis in total. The features of the datasets involved in this study are summarized in Supplementary Table S1. Given that these three microarray datasets derived from the same platform, gene expression datasets were merged into one expression matrix. R package *sva* was employed to eliminate the potential batch effect (Leek et al., 2012). Raw data was preprocessed identically with the R package *affy* by using the Robust Multichip Average (RMA) function for background correction and quantile normalization (Irizarry et al., 2003). Moreover, disease states and sample traits were merged and transformed into binary variables. Probes and samples were checked to remove missing values. Sample clustering was based on the Euclidean distance calculated with variance stabilized expression levels.

### Construction of WGCNA network

In order to ensure a reliable WGCNA network, the gene expression profiles of samples were clustered, and the outliers were identified and removed. The functions softConnectivity and pickSoftThreshold from the R package *WGCNA* (Langfelder and Horvath, 2008) were utilized to analyze the influence of power value on the scale independence and mean connectivity and build up a scale-free network. By calculating the scale-free topology fit index, the function pickSoftThreshold provided an appropriate soft-thresholding power for network construction. Meanwhile, the function softConnectivity calculated different mean connectivity of different soft thresholds. If the scale-free topology fit index values reached 0.9 for low powers (<30), it indicated that the topology of the network was scale-free (Liu et al., 2017; Li et al., 2020). With an appropriate soft threshold, an adjacency matrix was calculated, which was transformed into a topological overlap matrix (TOM) (Dong and Horvath, 2007). Subsequently, the corresponding dissimilarity (1-TOM) was calculated, which was beneficial to accomplishing a hierarchical clustering dendrogram. Then, a hierarchical clustering tree of genes was calculated by TOM. According to a parameter set up as minModuleSize (n = 30), modules were identified by the Dynamic Tree Cut algorithm.

To identify the OA-related modules, the next step was to integrate highly related modules. Module eigengene, the first principal component of a given module, was calculated for each module, which was considered as the representative of this module in a one-dimensional vector. Hierarchical cluster analysis was performed on all modules, and highly associated modules were merged by the Merged Dynamic algorithm. Module-trait associations were calculated by module eigengenes. The module-trait associations revealed the relationships between OA and modules by calculating the correlation coefficient and one-way ANOVA (Li et al., 2020; Cheng et al., 2021). Significant modules were identified according to the correlation coefficient and *p*-value. *p*-value < 0.05 was considered statistically significant.

### Function enrichment analysis and hub gene identification

ClusterProfiler was engaged for the Gene Ontology (GO) and Kyoto Encyclopedia of Genes and Genomes (KEGG) (Tang et al., 2018). GO consists of three aspects: biological process, molecular function, and cellular component. KEGG includes four databases: pathway, genes, compound, and enzyme. The GO terms and KEGG pathway with *p*-value adjusted by Benjamini and Hochberg method less than 0.05 (p.adjust < 0.05) were considered significant. The results of enrichments were visualized by bubble plot via the function *dotplot* in ClusterProfiler. The relationship between genes and pathways was visualized by heatmap plot via the function *heatplot* in ClusterProfiler. Hub genes were defined as the genes with high connectivity in the co-expressing network, potentially playing a critical role in the module. The hub genes were identified by the Cytoscape plugin cytoHubba (Chin et al., 2014). Two independent datasets GSE143514 (Zhao et al., 2021) and GSE12021 (Huber et al., 2008) were used to validate the expression of hub genes. The feature of the independent datasets is summarized in Supplementary Table S1.

### Prediction of the potential drug targeting modules

Hub genes derived from the significant modules were input into the CMap database (Lamb et al., 2006). Subsequently, the enrichment score that represents the similarities between the expression profiles of cells cultured with compounds and hub genes was estimated, and the compounds listed by enrichment score were revealed. Using the criteria of *p*-value < 0.01 and enrichment score <0, we identified the compounds that were significantly negatively correlated with hub genes.

## Result

### Data preprocessing, gene expression matrix, and weighted gene co-expression network

**FIGURE 2:**
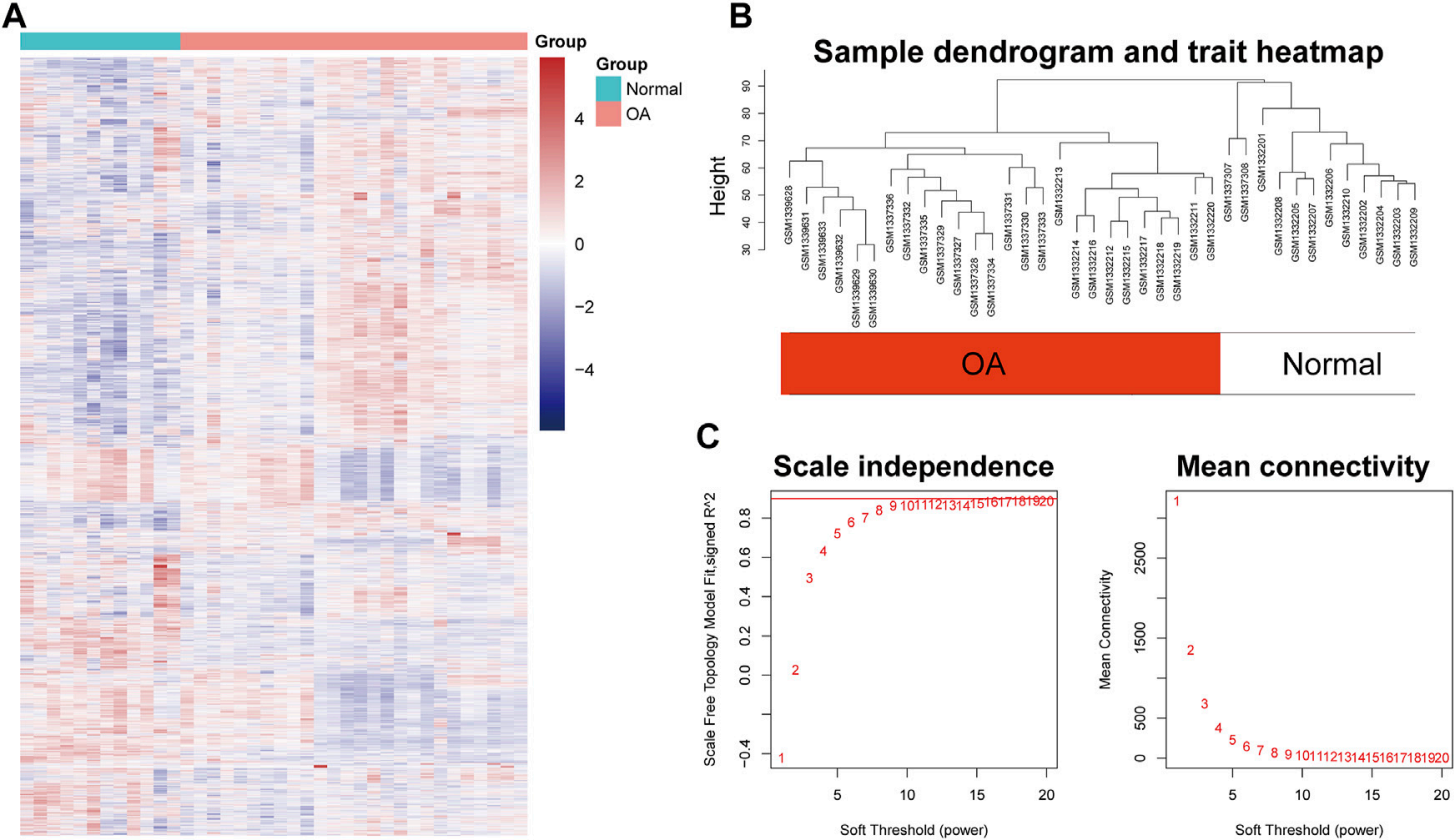
Sample clustering and soft-thresholding power determination. **(A)** Heatmap of the genes involved in WGCNA. **(B)** Clustering dendrogram of the gene expression of the synovial tissue from 26 OA patients and 12 healthy donors after excluding the outliers. Clustering was based on Euclidean distance calculated with variance stabilized expression level. **(C)** Analysis of scale independence and mean connectivity of the gene network at various soft-thresholding powers to determine the scale-free fit index.

**FIGURE 3:**
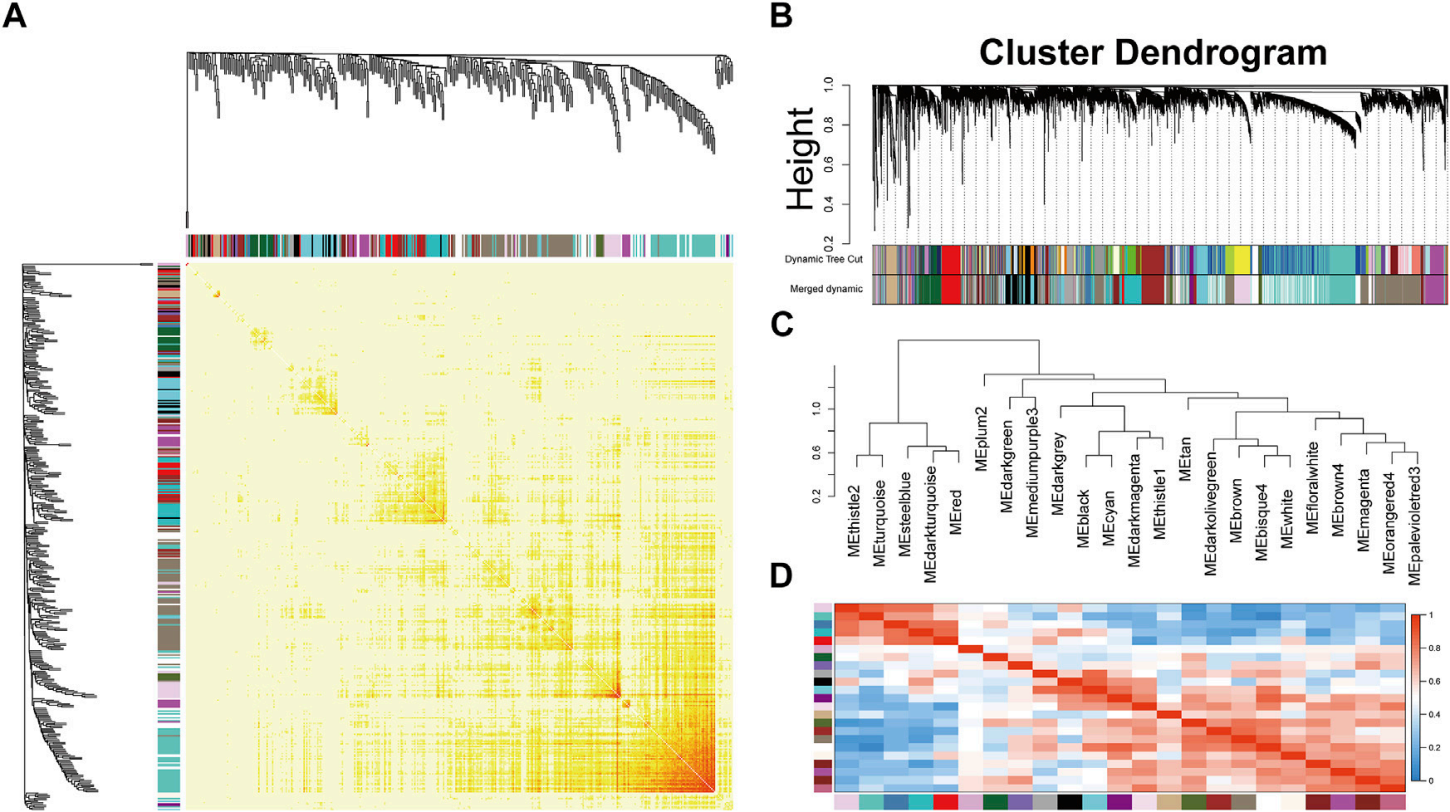
Topological overlap calculation and module identification. **(A)** Heatmap plot of topological overlap in the gene network. Red squares along the diagonal correspond to modules. **(B)** Gene dendrogram calculated by average linkage hierarchical clustering. The color row underneath the dendrogram shows the assigned original module and the merged module. **(C)** Hierarchical clustering of module eigengenes. **(D)** Heatmap plot of the eigengene adjacencies. Each row and column in the heatmap correspond to one module eigengene.

After preprocessing, the gene expression matrix of the top 50% probes in the rank of variance was obtained. According to the result of sample hierarchical clustering, potential outliers were detected and removed from the analysis, which involve a total of eight normal samples (GSM1337304, GSM 1337309, GSM133706, GSM1337310, GSM1337305, GSM1337313, GSM1337311, and GSM1337312) (Supplementary Figure S1). The gene expression of the remaining samples was visualized by a heatmap (Figure 2A). The sample dendrogram and trait heatmap were demonstrated in Figure 2B. The sample cluster revealed a high correlation between clinical traits (OA in red color and normal in white color) and gene expression profiles after excluding the outliers. We calculated the absolute value of the correlation coefficient of the top 50% probes in the rank of variance. Based on the correlation coefficient, the functions softConnectivity and pickSoftThreshold provided respective scale-free network module index and mean connectivity of different soft thresholds. As shown in Figure 2C, the soft-threshold power of 9 was the lowest power that met the requirements of the value of scale-free network module index >0.9 and sufficient mean connectivity. Based on this soft threshold, a scale-free network was built up and an adjacency matrix was computed and converted into a topological overlap matrix (TOM) (Figure 3A). We constructed a hierarchical clustering dendrogram of genes and identified 54 modules by the Dynamic Tree Cut algorithm (Figure 3B) that were further merged into 23 modules to reduce the module number (Figure 3C). These 23 modules were aggregated into two clusters that include five modules and 18 modules, respectively. The adjacency matrix of modules is visualized as a heatmap plot (Figure 3D).

### Critical modules, pathways, and hub genes of OA

**FIGURE 4:**
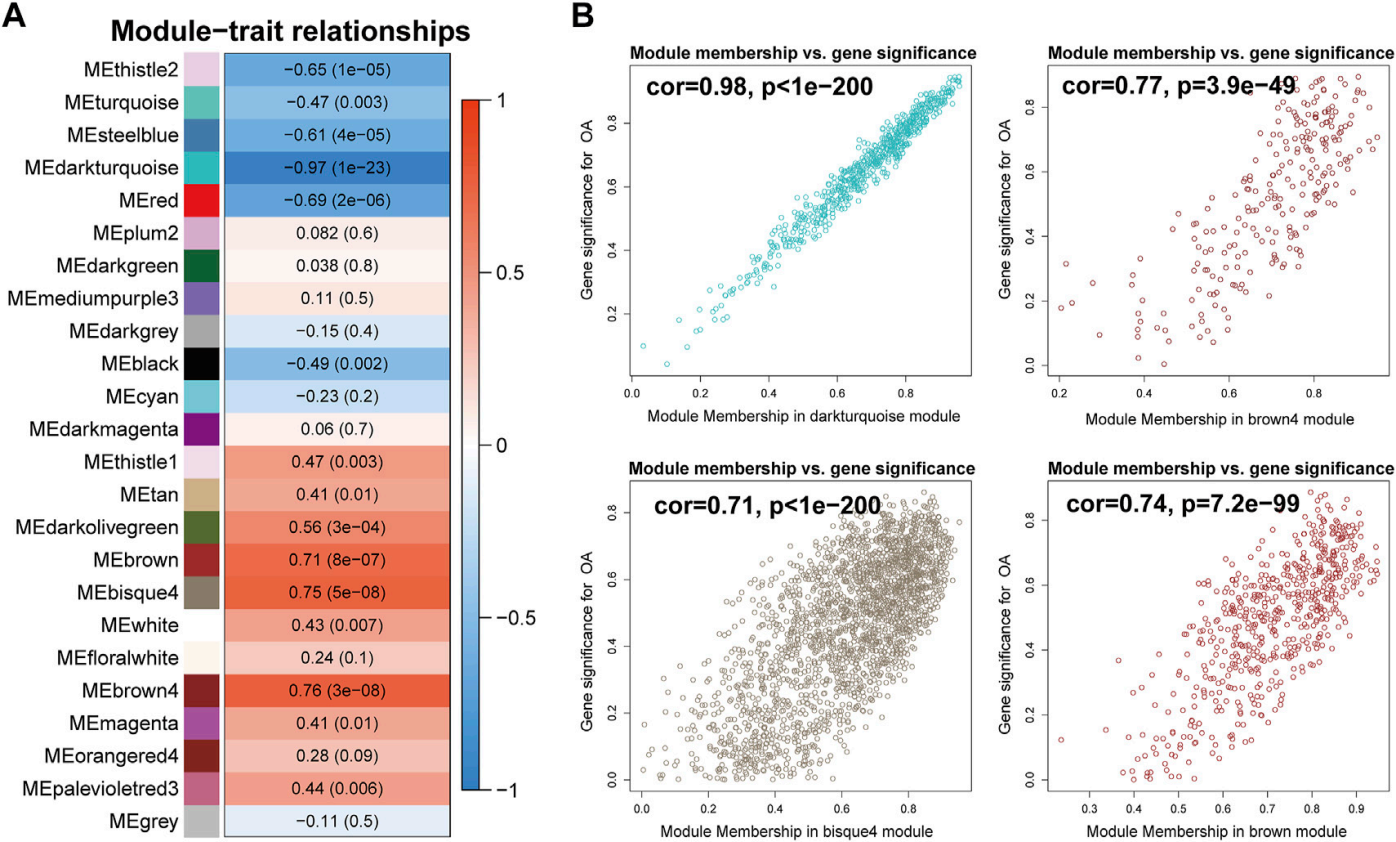
Identification of modules associated with rheumatoid arthritis. **(A)** Heatmap of the correlations between module eigengenes and OA. The value in each square and the p-value in parentheses reflect the correlation coefficient between the module eigengene and disease. **(B)** Scatter plot of the correlation between gene significance (GS) and gene module membership (MM) in OA-related modules, including the darkturquoise, the brown4, the bisque4, and the brown module.

To identify the biologically meaningful modules, the module-trait relationships were obtained by calculating the correlation coefficient and *p*-value between each module eigengene and sample traits. The modules with the absolute value of correlation coefficient >0.7 and *p*-value <0.05 were selected as significant modules. As shown in Figure 4A, the darkturquoise module (cor = -0.97, *p*-value = 1e-23) had the highest correlation with OA. Moreover, the brown4 module (cor = 0.76, *p*-value = 3e-08), the bisque4 module (cor = 0.75, *p*-value = 5e-08), and the brown module (cor = 0.71, *p*-value = 8e-07) were also highly related to OA. The genes in these modules also exhibited a good correlation between gene significance (GS) and gene module membership (MM), indicating that they were highly correlated to the corresponding module as well as OA (Figure 4B). To understand the biological relevance of these four modules to OA, GO term enrichment and KEGG pathway enrichment analyses were utilized to annotate the functions of 670 genes in the darkturquoise module, the 237 genes in the brown4 module, the 2,120 genes in the bisque4 module, and the 561 genes in the brown module.

**FIGURE 5:**
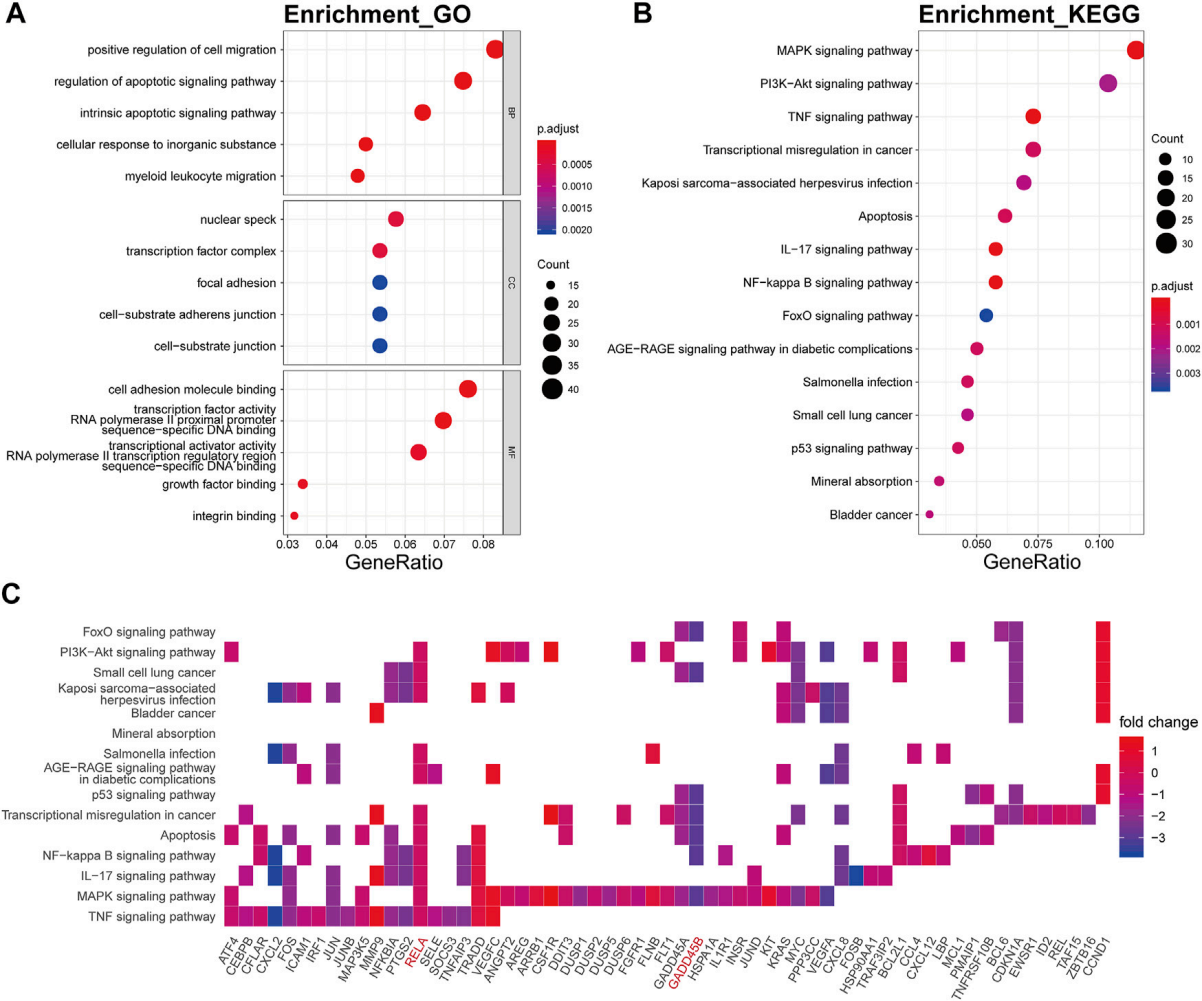
Functional annotation of the darkturquoise module. **(A)** GO term enrichment and **(B)** KEGG pathway analysis of the darkturquoise module. Gene ratio indicates the ratio of the enriched genes in each GO function and KEGG pathway. The color and size of the dot represent p-value adjusted by Benjamini and Hochberg method and the gene number assigned to the corresponding GO term and KEGG pathway, respectively. Functional annotation reveals critical signaling pathways in OA, such as MAPK, TNF, PI3K/Akt, NF-kB, and IL-17 signaling. **(C)** Heatmap plot of the connectivity of the enriched genes and KEGG pathways.

The biological processes of the darkturquoise module were relevant to positive regulation of cell migration (p.adjust = 6.61E-10), respond to regulation of apoptotic signaling pathway (p.adjust = 1.32E-10), respond to intrinsic apoptotic signaling pathway (p.adjust = 1.09E-10), respond to cellular response to inorganic substance (p.adjust = 1.60E-10), and respond to myeloid leukocyte migration (p.adjust = 6.48E-10) (Figure 5A). The KEGG pathway enrichment of the darkturquoise module was found to include mainly MAPK signaling pathway (p.adjust = 1.26E-07), PI3K-Akt signaling pathway (p.adjust = 1.14E-04), TNF signaling pathway (p.adjust = 6.92E-09), and transcriptional misregulation in cancer (p.adjust = 2.70E-05) (Figure 5B). The *RELA* gene is mostly involved in the KEGG enrichment pathway with high fold change values in the darkturquoise module (Figure 5C).

**FIGURE 6:**
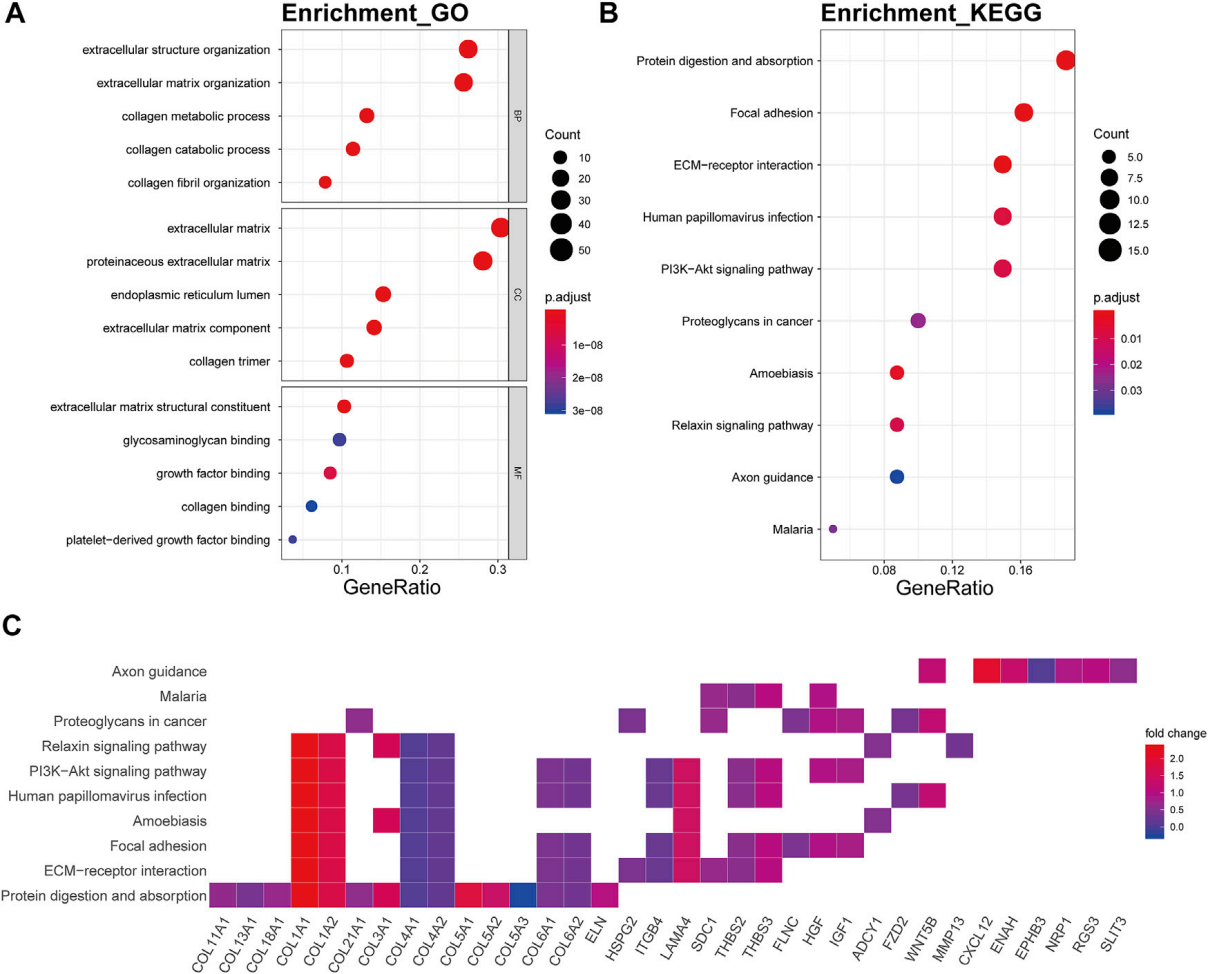
Functional annotation of the brown4 module. **(A)** GO term enrichment and **(B)** KEGG pathway analysis of the brown4 module. Gene ratio indicates the ratio of the enriched genes in each GO function and KEGG pathway. The color and size of the dot represent p-value adjusted by Benjamini and Hochberg method and the gene number assigned to the corresponding GO term and KEGG pathway, respectively. Functional annotation indicates the mechanism of ECM remodeling in OA. **(C)** Heatmap plot of the connectivity of the enriched genes and KEGG pathways.

The genes of the brown4 module were enriched in the extracellular matrix-related biological processes of extracellular structure organization (p.adjust = 2.06E-34), extracellular matrix organization (p.adjust = 6.23E-36), collagen metabolic (p.adjust = 6.53E-22), collagen catabolic (p.adjust = 3.90E-23), and collagen fibril organization (p.adjust = 4.08E-16) (Figure 6A). The genes were also enriched in several signaling pathway including protein digestion and absorption (p.adjust = 2.10E-14), focal adhesion (p.adjust = 1.76E-07), ECM-receptor interaction (p.adjust = 5.14E-11), and the PI3K-Akt signaling pathway (p.adjust = 3.91E-04) (Figure 6B). Collagen genes, such as COL1A1, COL1A2, COL4A1, and COL4A2, showed a good involvement to the KEGG enrichment pathway (Figure 6C).

**FIGURE 7:**
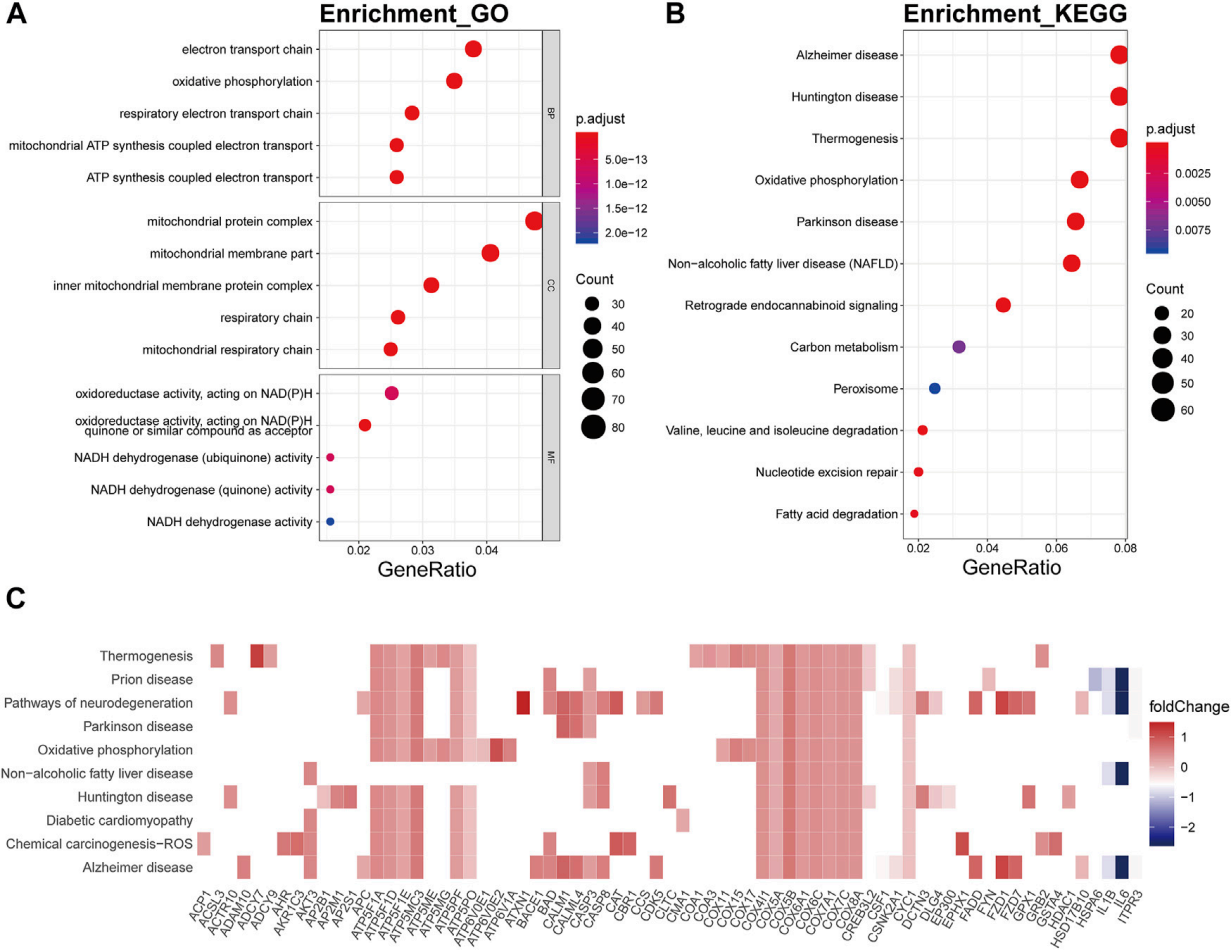
Functional annotation of the bisque4 module. **(A)** GO term enrichment and **(B)** KEGG pathway analysis of the bisque4 module. Gene ratio indicates the ratio of the enriched genes in each GO function and KEGG pathway. The color and size of the dot represent p-value adjusted by Benjamini and Hochberg method and the gene number assigned to the corresponding GO term and KEGG pathway, respectively. Functional annotation indicates the hallmarks of OA, such as mitochondrial dysfunction and altered energy metabolism. **(C)** Heatmap plot of the connectivity of the enriched genes and KEGG pathways.

In the bisque4 module, the biological processes involved mainly electron transport chain (p.adjust = 5.25E-21), oxidative phosphorylation (p.adjust = 2.65E-25), respiratory electron transport chain (p.adjust = 1.49E-19), mitochondrial ATP synthesis coupled electron transport (p.adjust = 1.02E-19), and ATP synthesis coupled electron transport (p.adjust = 1.68E-19) (Figure 7A). In KEGG analysis, these genes were relevant to the disease or pathway relating to aging, involving Alzheimer’s disease (p.adjust = 4.16E-21), Huntington’s disease (p.adjust = 8.23E-18), thermogenesis (p.adjust = 1.31E-13), oxidative phosphorylation (p.adjust = 2.46E-20), and Parkinson’s disease (p.adjust = 5.77E-18) (Figure 7B). The genes encoding ATP synthase subunits, such as ATP5F1A, ATP5F1D, ATP5F1E, ATP5MC3, ATP5PF, and ATP5PO, and cytochrome c oxidases, including COX5A, COX4I1, and COX7C, had high connectivity with pathway related to the bisque4 module, especially oxidative phosphorylation (Figure 7C).

**FIGURE 8:**
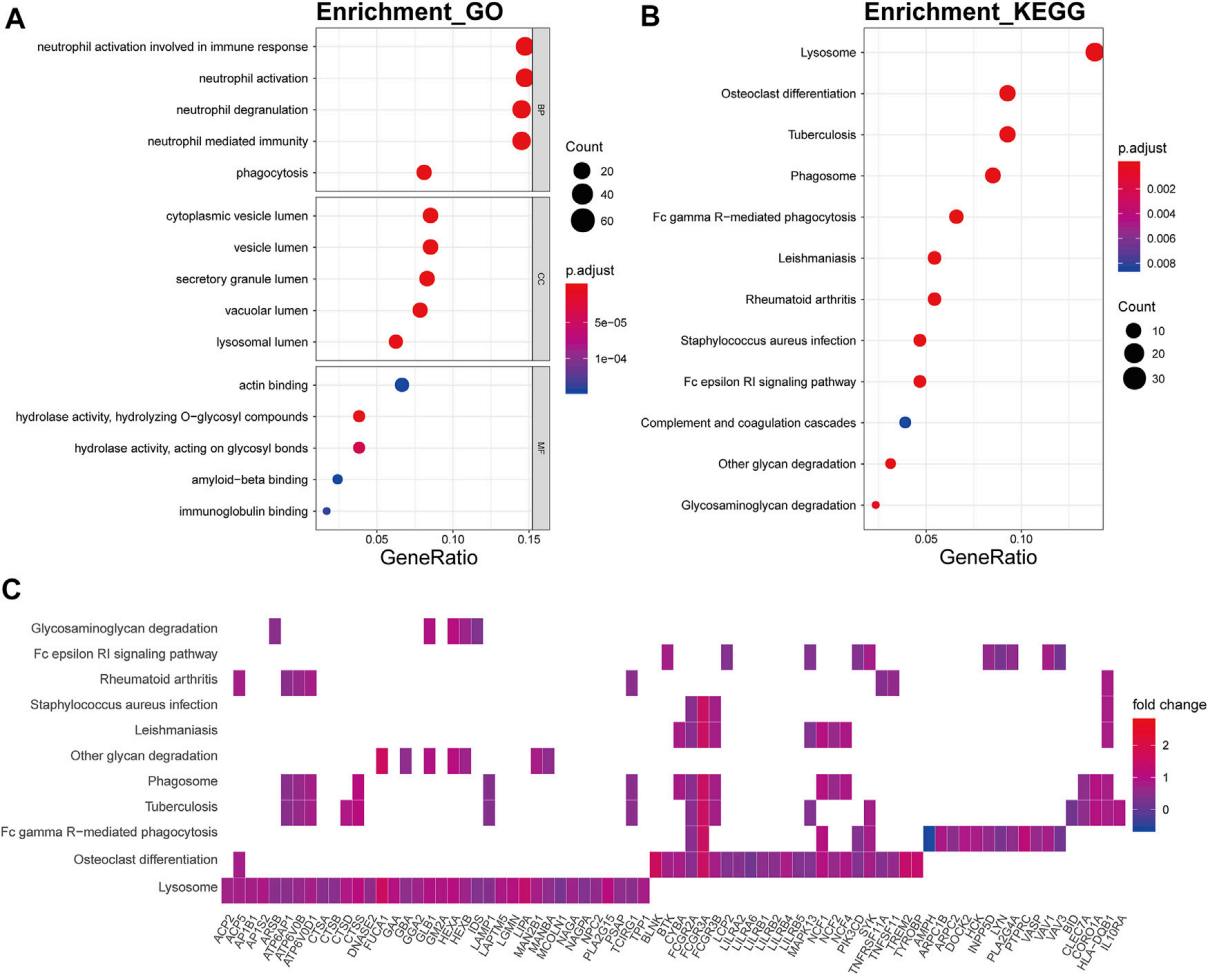
Functional annotation of the brown module. **(A)** GO term enrichment and **(B)** KEGG pathway analysis of the brown module. Gene ratio indicates the ratio of the enriched genes in each GO function and KEGG pathway. The color and size of the dot represent p-value adjusted by Benjamini and Hochberg method and the gene number assigned to the corresponding GO term and KEGG pathway, respectively. Functional annotation indicates the immune involvement in the OA mechanism. **(C)** Heatmap plot of the connectivity of the enriched genes and KEGG pathways.

The brown module was mainly involved in immune-related biological processes including neutrophil activation involved in immune respond, neutrophil activation (p.adjust = 8.14E-28), neutrophil degranulation (p.adjust = 1.20E-27), neutrophil mediated immunity (p.adjust = 6.73E-27), and phagocytosis (p.adjust = 4.74E-13) (Figure 8A). In KEGG pathway analysis, the brown module showed strong relevance to lysosome (p.adjust = 4.09E-24), osteoclast differentiation (p.adjust = 9.80E-12), tuberculosis (p.adjust = 1.23E-08), phagosome (p.adjust = 1.23E-08), and Fc gamma R-mediated phagocytosis (p.adjust = 1.23E-08) (Figure 8B). ATPase H^+^ transporters, including ATP6AP1, ATP6V0B, and ATP6V0D1, were identified in the other module-related pathways such as lysosome, tuberculosis, and phagosome (Figure 8C). Fc fragments of IgG receptors, such as FCGR3A, FCGR2A, and FCGR2B, were involved in osteoclast differentiation.

**FIGURE 9:**
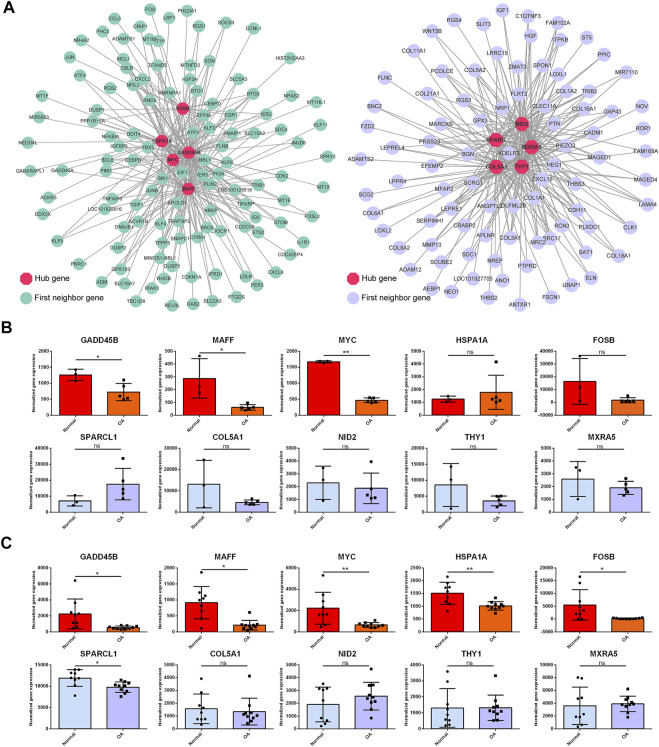
Topological analysis of the gene network and hub gene identification. **(A)** Gene network is based on WGCNA of the darkturquoise module and the brown4 module. Nodes and edges represent the genes and the connection between them. The hub genes (red) with the top five connectivity in the network are highlighted. The first neighbor genes are the genes co-expressed with the hub genes. Two independent datasets were employed to validate the hub genes **(B, C)**. GADD45B, MAFF, and MYC were validated as the downregulated genes in both datasets. OA versus normal; ns, not significant; *, *p* < 0.05; **, *p* < 0.01; all p-value estimated by unpaired Student’s t-test.

To identify the critical hub genes in the module, we constructed the co-expressing network of gene expression by WGCNA and visualized it by Cytoscape software (Figure 9A). Then, the genes with the top five connectivity, which was calculated by the cytoHubba plugin of Cytoscape, were defined as the hub genes in the module. As shown in Figure 9A, GADD45B, MAFF, MYC, HSPA1A, and FOSB were the hub genes in the darkturquoise module. For the brown4 module, SPARCL1, COL5A1, NID2, THY1, and MXRA5 were identified as the hub genes. Moreover, these genes were partially validated by an independent dataset (Figures 9B, C). Among the identified hub genes, GADD45B, MAFF, and MYC were validated as the downregulated genes.

### Potential drug targeting the significant module

To identify the potential drugs targeting the darkturquoise and the brown4 modules, the upregulated and downregulated genes were input into the CMap database. As shown in Table 1 and Table 2 the top 10 potential drugs based on the enrichment score were identified, respectively. Interestingly, anisomycin and MG-262 were identified as two common compounds targeting these modules, suggesting that they might be the promising drugs for OA.

**TABLE 1:** Potential drugs targeting the darkturquoise module.

Compound	Enrichment score^a^	Specificity^b^	Cell line^c^
Anisomycin	−0.99	0	MCF7, PC3, and HL60
MG-262	−0.98	0	MCF7 and PC3
Cicloheximide	−0.93	0.0148	MCF7, PC3, and HL60
Ikarugamycin	−0.906	0.0076	MCF7
5279552	−0.872	0.0238	MCF7
Isoflupredone	−0.864	0.125	MCF7, PC3, and HL60
Fasudil	−0.854	0.0056	MCF7 and PC3
Lomustine	−0.831	0.0141	MCF7 and PC3
Sulmazole	−0.823	0.0396	MCF7, PC3, and HL60
Lycorine	−0.808	0.0267	MCF7, PC3, and HL60

a Enrichment scores indicate the similarity of the gene expression of OA, module, and the gene signature of the compounds. Negative scores represent the opposite relationships between them.

b Specificity measures the uniqueness of the connection between a perturbagen and the signature of interest. Small values of specificity indicate high uniqueness between signatures and gene modules.

c Cell lines involved in the assessments.

**TABLE 2:** Potential drugs targeting the brown4 module.

Compound	Enrichment score ^a^	Specificity ^b^	Cell line ^c^
Anisomycin	−0.99	0	MCF7, PC3, and HL60
MG-262	−0.98	0	MCF7 and PC3
Cicloheximide	−0.93	0.0148	MCF7, PC3, and HL60
Ikarugamycin	−0.906	0.0076	MCF7
5279552	−0.872	0.0238	MCF7
Isoflupredone	−0.864	0.125	MCF7, PC3, and HL60
Fasudil	−0.854	0.0056	MCF7 and PC3
Lomustine	−0.831	0.0141	MCF7 and PC3
Sulmazole	−0.823	0.0396	MCF7, PC3, and HL60
Lycorine	−0.808	0.0267	MCF7, PC3, and HL60

a Enrichment scores indicate the similarity of the gene expression of OA, module, and the gene signature of the compounds. Negative scores represent the opposite relationships between them.

b Specificity measures the uniqueness of the connection between a perturbagen and the signature of interest. Small values of specificity indicate high uniqueness between signatures and gene modules.

c Cell lines involved in the assessments.

## Discussion

In this study, we employed integrated bioinformatics methods to identify the significant OA-related modules, signaling pathways, and critical hub genes. Among these modules, the darkturquoise module and the brown4 module should be highlighted based on their strong biological relevance to OA.

The pathway analysis of the darkturquoise module indicates the contributions of some important signaling pathways to OA, such as the MAPK signaling pathway, TNF signaling pathway, and PI3K/Akt signaling pathway. The roles of these pathways have been well characterized in OA (Grunke and Schulze-Koops, 2006; Loeser et al., 2008; Sun et al., 2020), including promoting chondrocyte apoptosis, inducing pro-inflammatory factors, increasing catabolic metabolism, or/and reducing anabolic metabolism individually or in a complicated crosstalk between these pathways. The most important finding should be addressed to the identification of *RELA* as a broadly-involved gene among the pathway relevant to this module. *RELA* encodes the subunit of NF-kB, namely, RelA or P65. *RELA* can be triggered by a host of stress-related stimuli including pro-inflammatory cytokines, activating NF-kB signaling, and promoting catabolism (Marcu et al., 2010). Moreover, GADD45B was identified as a critical hub gene in the darkturquoise module. GADD45B is characterized as a regulator implicated in a variety of responses to cell injury including cell cycle checkpoints, apoptosis, and DNA repair (Salvador et al., 2013). Decreased GADD45B was found in the cartilage of patients with late-stage OA (Ijiri et al., 2008). Silencing GADD45B decreased chondrocyte survival and enhanced apoptosis induced by TNF-alpha (Ijiri et al., 2008). These lines of evidence indicate a protective effect of GADD45B in chondrocytes. Moreover, GADD45B deficiency can contribute to the activation of JNK and elevated MMP3 and MMP13 gene expression in fibroblast-like synoviocytes (FLS) (Svensson et al., 2009). Since GADD45B was identified as a hub gene in our study, it may be an important gene in the mechanisms of OA development and a promising target for OA. Moreover, MAFF and MYC are the other two hub genes in this module. MAFF, one of the basic region leucine zipper (bZIP)-type transcription factors, participates in transcriptional activation or repression (Katsuoka and Yamamoto, 2016). MAFF is identified as an oncogene that regulates IL11/STAT3 signaling (Moon et al., 2021). Moreover, MAFF is a link between inflammation, lipid, and lipoprotein metabolism (von Scheidt et al., 2021). These lines of evidence suggest that MAFF may be a regulator of inflammation in OA. Indeed, although the roles of MAFF have not been documented in OA, Garcia et al. found that MAFF is hypermethylated and downregulated in OA cartilage (Alvarez-Garcia et al., 2016). MYC is a well-documented transcription factor that is extensively involved in stem cell biology cancer (Dang et al., 2006; Yoshida, 2018). Fisch et al. (2018) found that JUN, EGR1, JUND, FOSL2, MYC, KLF4, RELA, and FOS both target large numbers of dysregulated genes in OA and are themselves suppressed in OA.

The other module also revealed some critical pathways and genes relevant to OA. In the brown4 module, GO and KEGG functional analysis indicated the involvement of the alteration of the extracellular matrix (ECM). The inflammatory factors, including IL-1β, TNF-α, IL-12, and IL-15, increased the expression of matrix-degrading proteins such as matrix metalloproteinases (MMPs) (Maldonado and Nam, 2013). During OA, the content of aggrecan, a negatively charged proteoglycan that attracts water molecules, decreased in patients’ tissue. Collagen type II, distributed in the normal healthy cartilage matrix and providing tensile support for the tissue, is transformed into collagen type I (Goldring and Goldring, 2010; Lahm et al., 2010; Zhu et al., 2013). Reduced collagen type II decreased stored elastic energy and gave rise to fibrillation and fissure formation (Silver et al., 2002). The alteration of ECM is tightly associated with ECM–receptor interaction and focal adhesion. Some studies reported that the expression of collagen V (COL5A1) was increased in OA cartilage (Wei et al., 2010), however, the detailed function of COL5A1 remains to be demonstrated. The pathway analysis of the bisque4 module revealed the hallmarks of OA and aging, such as mitochondrial dysfunctions and abnormal energy metabolism (Wei et al., 2010). In the brown module, the immune-relevant pathways and genes were indicated. The roles of complement activation and pro-inflammatory cytokines in cartilage destruction and synovitis were well documented (Kalaitzoglou et al., 2017; Lopes et al., 2017). Also, the infiltration of immune cells, such as T cells and activated macrophages in the OA synovial tissue, may be an important event relevant to low-grade inflammation and pain.

Except for the exploration of the OA-relevant genes and signaling to advance the understanding of the OA mechanism, some potential compounds targeting these genes were also identified based on the CMap database. The most interesting finding is that two compounds, anisomycin and MG-262, were predicted to target both the darkturquoise and the brown4 module. Anisomycin, as a P38 agonist, increased the gene expression of COL2A1 and decreased the gene expression of COL10A1, resulting in the inhibition of chondrocyte hypertrophy (Li et al., 2010). Moreover, p38 activation stabilized SOX9 mRNA (Tew and Hardingham, 2006), suggesting that anisomycin might be able to increase SOX9 expression by activating P38 to promote anabolic metabolism. MG-262, as a proteasome inhibitor, was shown to inhibit IL-1β/TNF-α-induced activation of NF-κB, indicating the potential of MG-262 in attenuating OA by the inhibition of the NF-κB signaling pathway (Pujols et al., 2012). Still, the effects of anisomycin and MG-262 on OA remain elusive because they are likely to be toxic and aggravate OA progression at high concentrations.

The limitations of this study should be discussed. First, we identified key OA-related hub genes and only validated a few of them by an independent dataset. Second, no experimental exploration was included to reveal the role of these key genes. Finally, the compounds we identified targeting to OA-related gene module were based on estimation of the other cell lines. Further experiential studies are needed to verify our findings in the future.

## Conclusion

The significant modules, signaling pathways, and potential hub genes relevant to OA were identified in this study. Anisomycin and MG-262 might be the potential drugs for OA therapy.

## Data Availability

The datasets presented in this study can be found in online repositories. The names of the repository/repositories and accession number(s) can be found in the article/Supplementary Material.

## References

[B1] Altman R. D. Manjoo A. Fierlinger A. Niazi F. Nicholls M. (2015). The mechanism of action for hyaluronic acid treatment in the osteoarthritic knee: A systematic review. BMC Musculoskelet. Disord. 16, 321. 10.1186/s12891-015-0775-z 26503103 PMC4621876

[B2] Alvarez-Garcia O. Fisch K. M. Wineinger N. E. Akagi R. Saito M. Sasho T. (2016). Increased DNA methylation and reduced expression of transcription factors in human osteoarthritis cartilage. Arthritis Rheumatol. 68, 1876–1886. 10.1002/art.39643 26881698 PMC4963260

[B3] Cheng C. Zhou J. Chen R. Shibata Y. Tanaka R. Wang J. (2021). Predicted disease-specific immune infiltration patterns decode the potential mechanisms of long non-coding RNAs in primary sjogren's syndrome. Front. Immunol. 12, 624614. 10.3389/fimmu.2021.624614 33936039 PMC8079748

[B4] Chin C. H. Chen S. H. Wu H. H. Ho C. W. Ko M. T. Lin C. Y. (2014). cytoHubba: identifying hub objects and sub-networks from complex interactome. BMC Syst. Biol. 8 (Suppl. 4), S11. 10.1186/1752-0509-8-S4-S11 25521941 PMC4290687

[B5] Dang C. V. O'donnell K. A. Zeller K. I. Nguyen T. Osthus R. C. Li F. (2006). The c-Myc target gene network. Semin. Cancer Biol. 16, 253–264. 10.1016/j.semcancer.2006.07.014 16904903

[B6] Dong J. Horvath S. (2007). Understanding network concepts in modules. BMC Syst. Biol. 1, 24. 10.1186/1752-0509-1-24 17547772 PMC3238286

[B7] Douglas R. J. (2012). Corticosteroid injection into the osteoarthritic knee: Drug selection, dose, and injection frequency. Int. J. Clin. Pract. 66, 699–704. 10.1111/j.1742-1241.2012.02963.x 22698422

[B8] Fisch K. M. Gamini R. Alvarez-Garcia O. Akagi R. Saito M. Muramatsu Y. (2018). Identification of transcription factors responsible for dysregulated networks in human osteoarthritis cartilage by global gene expression analysis. Osteoarthr. Cartil. 26, 1531–1538. 10.1016/j.joca.2018.07.012 PMC6245598 30081074

[B9] Goldring M. B. Goldring S. R. (2010). Articular cartilage and subchondral bone in the pathogenesis of osteoarthritis. Ann. N. Y. Acad. Sci. 1192, 230–237. 10.1111/j.1749-6632.2009.05240.x 20392241

[B10] Grunke M. Schulze-Koops H. (2006). Successful treatment of inflammatory knee osteoarthritis with tumour necrosis factor blockade. Ann. Rheum. Dis. 65, 555–556. 10.1136/ard.2006.053272 16531556 PMC1798087

[B11] Huber R. Hummert C. Gausmann U. Pohlers D. Koczan D. Guthke R. (2008). Identification of intra-group, inter-individual, and gene-specific variances in mRNA expression profiles in the rheumatoid arthritis synovial membrane. Arthritis Res. Ther. 10, R98. 10.1186/ar2485 18721452 PMC2575612

[B12] Ijiri K. Zerbini L. F. Peng H. Otu H. H. Tsuchimochi K. Otero M. (2008). Differential expression of GADD45beta in normal and osteoarthritic cartilage: Potential role in homeostasis of articular chondrocytes. Arthritis Rheum. 58, 2075–2087. 10.1002/art.23504 18576389 PMC3950332

[B13] Irizarry R. A. Hobbs B. Collin F. Beazer-Barclay Y. D. Antonellis K. J. Scherf U. (2003). Exploration, normalization, and summaries of high density oligonucleotide array probe level data. Biostatistics 4, 249–264. 10.1093/biostatistics/4.2.249 12925520

[B14] Kalaitzoglou E. Griffin T. M. Humphrey M. B. (2017). Innate immune responses and osteoarthritis. Curr. Rheumatol. Rep. 19, 45. 10.1007/s11926-017-0672-6 28718060

[B15] Katsuoka F. Yamamoto M. (2016). Small maf proteins (MafF, MafG, MafK): History, structure and function. Gene 586, 197–205. 10.1016/j.gene.2016.03.058 27058431 PMC4911266

[B16] Kraus V. B. Blanco F. J. Englund M. Karsdal M. A. Lohmander L. S. (2015). Call for standardized definitions of osteoarthritis and risk stratification for clinical trials and clinical use. Osteoarthr. Cartil. 23, 1233–1241. 10.1016/j.joca.2015.03.036 PMC4516635 25865392

[B17] Lahm A. Mrosek E. Spank H. Erggelet C. Kasch R. Esser J. (2010). Changes in content and synthesis of collagen types and proteoglycans in osteoarthritis of the knee joint and comparison of quantitative analysis with Photoshop-based image analysis. Arch. Orthop. Trauma Surg. 130, 557–564. 10.1007/s00402-009-0981-y 19838720

[B18] Lamb J. Crawford E. D. Peck D. Modell J. W. Blat I. C. Wrobel M. J. (2006). The connectivity map: Using gene-expression signatures to connect small molecules, genes, and disease. Science 313, 1929–1935. 10.1126/science.1132939 17008526

[B19] Langfelder P. Horvath S. (2008). Wgcna: an R package for weighted correlation network analysis. BMC Bioinforma. 9, 559. 10.1186/1471-2105-9-559 PMC2631488 19114008

[B20] Leek J. T. Johnson W. E. Parker H. S. Jaffe A. E. Storey J. D. (2012). The sva package for removing batch effects and other unwanted variation in high-throughput experiments. Bioinformatics 28, 882–883. 10.1093/bioinformatics/bts034 22257669 PMC3307112

[B21] Li T. F. Gao L. Sheu T. J. Sampson E. R. Flick L. M. Konttinen Y. T. (2010). Aberrant hypertrophy in smad3-deficient murine chondrocytes is rescued by restoring transforming growth factor beta-activated kinase 1/activating transcription factor 2 signaling: A potential clinical implication for osteoarthritis. Arthritis Rheum. 62, 2359–2369. 10.1002/art.27537 20506210 PMC2921996

[B22] Li X. Yang Y. Sun G. Dai W. Jie X. Du Y. (2020). Promising targets and drugs in rheumatoid arthritis: A module-based and cumulatively scoring approach. Bone Jt. Res. 9, 501–514. 10.1302/2046-3758.98.BJR-2019-0301.R1 PMC7468554 32922758

[B23] Liu C. Y. Li C. D. Wang L. Ren S. Yu F. B. Li J. G. (2018). Function scores of different surgeries in the treatment of knee osteoarthritis: A PRISMA-compliant systematic review and network-meta analysis. Med. Baltim. 97, e10828. 10.1097/MD.0000000000010828 PMC6393067 29794771

[B24] Liu X. Hu A. X. Zhao J. L. Chen F. L. (2017). Identification of key gene modules in human osteosarcoma by Co-expression analysis weighted gene Co-expression network analysis (WGCNA). J. Cell. Biochem. 118, 3953–3959. 10.1002/jcb.26050 28398605

[B25] Loeser R. F. Erickson E. A. Long D. L. (2008). Mitogen-activated protein kinases as therapeutic targets in osteoarthritis. Curr. Opin. Rheumatol. 20, 581–586. 10.1097/BOR.0b013e3283090463 18698181 PMC2892710

[B26] Lopes E. B. P. Filiberti A. Husain S. A. Humphrey M. B. (2017). Immune contributions to osteoarthritis. Curr. Osteoporos. Rep. 15, 593–600. 10.1007/s11914-017-0411-y 29098574

[B27] Mahmoudian A. Lohmander L. S. Mobasheri A. Englund M. Luyten F. P. (2021). Early-stage symptomatic osteoarthritis of the knee - time for action. Nat. Rev. Rheumatol. 17, 621–632. 10.1038/s41584-021-00673-4 34465902

[B28] Maldonado M. Nam J. (2013). The role of changes in extracellular matrix of cartilage in the presence of inflammation on the pathology of osteoarthritis. Biomed. Res. Int. 2013, 284873. 10.1155/2013/284873 24069595 PMC3771246

[B29] Marcu K. B. Otero M. Olivotto E. Borzi R. M. Goldring M. B. (2010). NF-kappaB signaling: Multiple angles to target OA. Curr. Drug Targets 11, 599–613. 10.2174/138945010791011938 20199390 PMC3076145

[B30] Maudens P. Jordan O. Allemann E. (2018). Recent advances in intra-articular drug delivery systems for osteoarthritis therapy. Drug Discov. Today 23, 1761–1775. 10.1016/j.drudis.2018.05.023 29792929

[B31] Moon E. J. Mello S. S. Li C. G. Chi J. T. Thakkar K. Kirkland J. G. (2021). The HIF target MAFF promotes tumor invasion and metastasis through IL11 and STAT3 signaling. Nat. Commun. 12, 4308. 10.1038/s41467-021-24631-6 34262028 PMC8280233

[B32] Pujols L. Fernandez-Bertolin L. Fuentes-Prado M. Alobid I. Roca-Ferrer J. Agell N. (2012). Proteasome inhibition reduces proliferation, collagen expression, and inflammatory cytokine production in nasal mucosa and polyp fibroblasts. J. Pharmacol. Exp. Ther. 343, 184–197. 10.1124/jpet.111.190710 22787116

[B33] Qu X. A. Rajpal D. K. (2012). Applications of Connectivity Map in drug discovery and development. Drug Discov. Today 17, 1289–1298. 10.1016/j.drudis.2012.07.017 22889966

[B34] Salvador J. M. Brown-Clay J. D. Fornace A. J. Jr. (2013). Gadd45 in stress signaling, cell cycle control, and apoptosis. Adv. Exp. Med. Biol. 793, 1–19. 10.1007/978-1-4614-8289-5_1 24104470

[B35] Siebelt M. Agricola R. Weinans H. Kim Y. J. (2014). The role of imaging in early hip OA. Osteoarthr. Cartil. 22, 1470–1480. 10.1016/j.joca.2014.04.030 25278058

[B36] Silver F. H. Bradica G. Tria A. (2002). Elastic energy storage in human articular cartilage: Estimation of the elastic modulus for type II collagen and changes associated with osteoarthritis. Matrix Biol. 21, 129–137. 10.1016/s0945-053x(01)00195-0 11852229

[B37] Sun K. Luo J. Guo J. Yao X. Jing X. Guo F. (2020). The PI3K/AKT/mTOR signaling pathway in osteoarthritis: A narrative review. Osteoarthr. Cartil. 28, 400–409. 10.1016/j.joca.2020.02.027 32081707

[B38] Svensson C. I. Inoue T. Hammaker D. Fukushima A. Papa S. Franzoso G. (2009). Gadd45beta deficiency in rheumatoid arthritis: Enhanced synovitis through JNK signaling. Arthritis Rheum. 60, 3229–3240. 10.1002/art.24887 19877043 PMC2858378

[B39] Tang L. Cheng Y. Zhu C. Yang C. Liu L. Zhang Y. (2018). Integrative methylome and transcriptome analysis to dissect key biological pathways for psoriasis in Chinese Han population. J. Dermatol. Sci. 91, 285–291. 10.1016/j.jdermsci.2018.06.001 29914851

[B40] Tang X. Wang S. Zhan S. Niu J. Tao K. Zhang Y. (2016). The prevalence of symptomatic knee osteoarthritis in China: Results from the China Health and retirement longitudinal study. Arthritis Rheumatol. 68, 648–653. 10.1002/art.39465 26474054

[B41] Tew S. R. Hardingham T. E. (2006). Regulation of SOX9 mRNA in human articular chondrocytes involving p38 MAPK activation and mRNA stabilization. J. Biol. Chem. 281, 39471–39479. 10.1074/jbc.M604322200 17050539

[B42] Valdes A. M. Spector T. D. (2011). Genetic epidemiology of hip and knee osteoarthritis. Nat. Rev. Rheumatol. 7, 23–32. 10.1038/nrrheum.2010.191 21079645

[B43] Von Scheidt M. Zhao Y. De Aguiar Vallim T. Q. Che N. Wierer M. Seldin M. M. (2021). Transcription factor MAFF (MAF basic leucine zipper transcription factor F) regulates an atherosclerosis relevant network connecting inflammation and cholesterol metabolism. Circulation 143, 1809–1823. 10.1161/CIRCULATIONAHA.120.050186 33626882 PMC8124091

[B44] Wei T. Kulkarni N. H. Zeng Q. Q. Helvering L. M. Lin X. Lawrence F. (2010). Analysis of early changes in the articular cartilage transcriptisome in the rat meniscal tear model of osteoarthritis: Pathway comparisons with the rat anterior cruciate transection model and with human osteoarthritic cartilage. Osteoarthr. Cartil. 18, 992–1000. 10.1016/j.joca.2010.04.012 20434574

[B45] Woetzel D. Huber R. Kupfer P. Pohlers D. Pfaff M. Driesch D. (2014). Identification of rheumatoid arthritis and osteoarthritis patients by transcriptome-based rule set generation. Arthritis Res. Ther. 16, R84. 10.1186/ar4526 24690414 PMC4060460

[B46] Yang F. Hu H. Yin W. Li G. Yuan T. Xie X. (2018). Autophagy is independent of the chondroprotection induced by platelet-rich plasma releasate. Biomed. Res. Int. 2018, 9726703. 10.1155/2018/9726703 30140705 PMC6081522

[B47] Yin M. Zhang J. Zeng X. Zhang H. Gao Y. (2021). Target identification and drug discovery by data-driven hypothesis and experimental validation in ovarian endometriosis. Fertil. Steril. 116, 478–492. 10.1016/j.fertnstert.2021.01.027 33714537

[B48] Yoshida G. J. (2018). Emerging roles of Myc in stem cell biology and novel tumor therapies. J. Exp. Clin. Cancer Res. 37, 173. 10.1186/s13046-018-0835-y 30053872 PMC6062976

[B49] Zhang W. Moskowitz R. W. Nuki G. Abramson S. Altman R. D. Arden N. (2008). OARSI recommendations for the management of hip and knee osteoarthritis, Part II: OARSI evidence-based, expert consensus guidelines. Osteoarthr. Cartil. 16, 137–162. 10.1016/j.joca.2007.12.013 18279766

[B50] Zhang Y. Jordan J. M. (2010). Epidemiology of osteoarthritis. Clin. Geriatr. Med. 26, 355–369. 10.1016/j.cger.2010.03.001 20699159 PMC2920533

[B51] Zhao Y. Lv J. Zhang H. Xie J. Dai H. Zhang X. (2021). Gene expression profiles analyzed using integrating RNA sequencing, and microarray reveals increased inflammatory response, proliferation, and osteoclastogenesis in pigmented villonodular synovitis. Front. Immunol. 12, 665442. 10.3389/fimmu.2021.665442 34248943 PMC8264543

[B52] Zhu Y. Yuan M. Meng H. Y. Wang A. Y. Guo Q. Y. Wang Y. (2013). Basic science and clinical application of platelet-rich plasma for cartilage defects and osteoarthritis: A review. Osteoarthr. Cartil. 21, 1627–1637. 10.1016/j.joca.2013.07.017 23933379

