# Supplementary material for: Unbiased transcriptome mapping and modeling identify candidate genes and compounds of osteoarthritis
Source: Front Pharmacol. 2022 Aug 10;13:888533. doi: 10.3389/fphar.2022.888533 (PMC9399521; doi:10.3389/fphar.2022.888533)
Supplement: Supplementary file 1 [file Table1.DOCX]

| **GEO Accession** | **Platform** | **Sample Size** | **Reference** |
| --- | --- | --- | --- |
| GSE55235 | GPL96 | Healthy: 10; OA: 10 | Woetzel et al., 2014 |
| GSE55457 | GPL96 | Healthy: 10; OA: 10 | Woetzel et al., 2014 |
| GSE55584 | GPL96 | Healthy: 0; OA: 6 | Woetzel et al., 2014 |
| GSE143514 | GPL20795 | Healthy: 3; OA: 5 | Zhao et al., 2021 |
| GSE12021 | GPL96 | Healthy: 9; OA: 10 | Huber et al., 2018 |

**Supplemental Table 1. Summary of the datasets involved in this study.**
