# Supplementary figures and images for: Unbiased transcriptome mapping and modeling identify candidate genes and compounds of osteoarthritis
Source: Front Pharmacol. 2022 Aug 10;13:888533. doi: 10.3389/fphar.2022.888533 (PMC9399521; doi:10.3389/fphar.2022.888533)

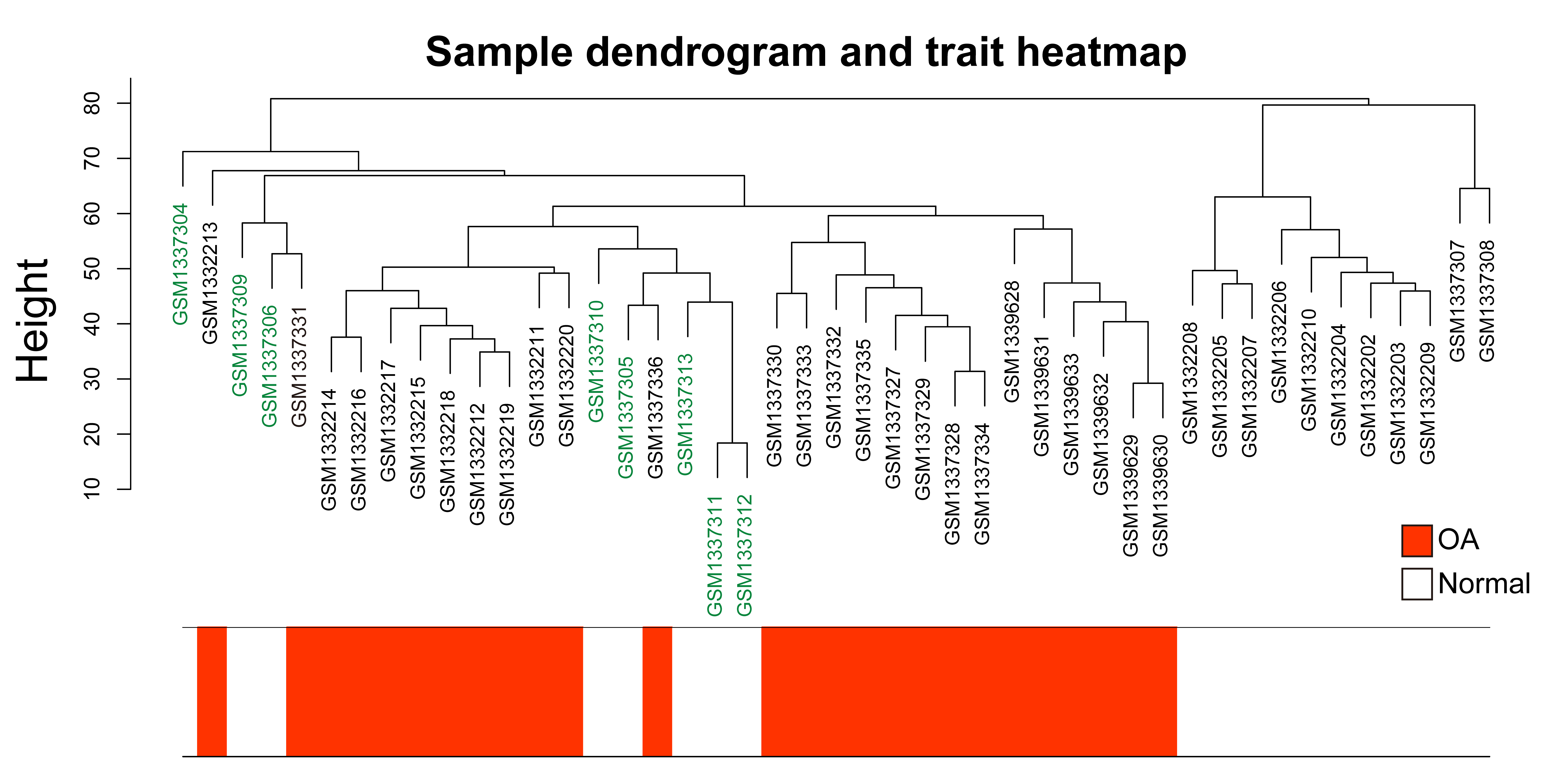

Supplement: Supplementary file 2 [file Image1.TIF]
